# Safety and Efficacy of Ambroxol Therapy in Polish Patients with Gaucher Disease

**DOI:** 10.3390/life16030485

**Published:** 2026-03-16

**Authors:** Patryk Lipiński, Dariusz Rokicki, Karolina Chwiałkowska, Michał Ciborowski, Joanna Godzień, Aleksandra Jezela-Stanek, Urszula Korotko, Mirosław Kwaśniewski, Magdalena Niemira, Paulina Szymańska-Rożek, Małgorzata Syczewska, Anna Tylki-Szymańska

**Affiliations:** 1 Department of Pediatrics, Medical Center of Postgraduate Education, 01-813 Warsaw, Poland; 2 Department of Pediatrics, Nutrition and Metabolic Diseases, The Children’s Memorial Health Institute, 04-730 Warsaw, Poland; d.rokicki@ipczd.pl; 3 Laboratory of Computational Molecular Medicine, Clinical Research Centre, Medical University of Białystok, 15-089 Białystok, Poland; karolina.chwialkowska@umb.edu.pl; 4 Metabolomics and Proteomics Laboratory, Clinical Research Centre, Medical University of Białystok, 15-089 Białystok, Poland; michal.ciborowski@umb.edu.pl (M.C.); joanna.godzien@umb.edu.pl (J.G.); 5 Department of Medical Biochemistry, Medical University of Białystok, 15-089 Białystok, Poland; 6 Department of Genetics and Clinical Immunology, National Institute of Tuberculosis and Lung Diseases, 01-138 Warsaw, Poland; jezela@gmail.com; 7 Centre for Digital Medicine, Medical University of Białystok, 15-089 Białystok, Poland; urszula.korotko@umb.edu.pl; 8 Centre for Bioinformatics and Data Analysis, Medical University of Białystok, 15-089 Białystok, Poland; miroslaw.kwasniewski@umb.edu.pl; 9 Laboratory of Genomics and Epigenetic Analysis, Clinical Research Centre, Medical University of Białystok, 15-089 Białystok, Poland; magdalena.niemira@umb.edu.pl; 10 Faculty of Mathematics, Informatics and Mechanics, University of Warsaw, 00-927 Warsaw, Poland; p.szymanska@gmail.com; 11 Department of Rehabilitation, The Children’s Memorial Health Institute, 04-730 Warsaw, Poland; m.syczewska@ipczd.pl

**Keywords:** Gaucher disease type 3, Gaucher disease neuronopathic form, neurological symptoms, ambroxol

## Abstract

Background: Gaucher disease (GD) is a lysosomal storage disorder caused by deficiency of β-glucocerebrosidase, leading to accumulation of glucocerebroside in lysosomes. Type 1 GD is most commonly associated with the N370S mutation and lacks neurological involvement, whereas the neuronopathic forms (types 2 and 3), frequently linked to L444P homozygosity, present with progressive neurological symptoms. Enzyme replacement therapy (ERT) effectively treats visceral manifestations but does not cross the blood–brain barrier and, therefore, does not improve neurological outcomes. Ambroxol, a plant-derived mucolytic agent, has been shown to act as a pharmacological chaperone capable of increasing residual enzyme activity and crossing into the central nervous system, with reports suggesting neurological benefit in L444P homozygotes. Methods: We evaluated 13 patients with type 3 GD (L444P/L444P homozygotes) who received ambroxol at 10 mg/kg/day for one year as part of a clinical trial. All participants had been on long-term ERT with stable biomarker levels (chitotriosidase, glucosylsphingosine [Lyso-GL1]) and hematological parameters. Neurological symptoms were assessed using the modified Severity Scoring Tool (mSST). Biomarkers and hematologic indices were monitored throughout the study. Results: Ambroxol treatment resulted in a reduction in severity or complete resolution of selected neurological symptoms in several patients. Conclusions: In patients with type 3 GD receiving stable ERT, ambroxol demonstrated beneficial effects on neurological symptom expression. Some improvement was observed in biomarkers; the activity of chitotrosidase and concentration of lyso-Gl1 decreased. These findings support the therapeutic potential of ambroxol as an adjunctive treatment for neuronopathic Gaucher disease.

## 1. Introduction

Gaucher disease (GD) is lysosomal storage disorder, classified within the group of sphingolipidoses. Its occurrence is pan-ethnic, meaning that GD is found among representatives of all ethnic groups, although the frequency of the disease varies. In the general population, the estimated incidence is approximately 1 in 40,000 to 1 in 60,000 live births. In the Caucasian population, a specific genetic variant—c.1226A>G, p.Asn409Ser (N307S)—is more frequent due to a founder effect. Particularly among Ashkenazi Jews, the incidence of GD may reach as high as 1 in 800 live births [1].

Due to a deficiency in β-glucocerebrosidase (GCase) activity, proper catabolism of glucocerebroside (into glucose and ceramide)—one of the main components of the cell membrane—is impaired. As a consequence of this deficiency, substrate (GlcCer) and its deacylated form—glycosylsphingosine (Lyso-Gb1)—accumulate primarily in macrophages, inducing their transformation into Gaucher cells.

Gaucher cells infiltrate mainly the spleen, liver, and bone marrow, but also other organs, and are considered the primary drivers of the clinical manifestations of the disease. Intensive substrate accumulation occurs in macrophages as a result of endocytosis of degraded blood cells, whose membranes are rich in glucocerebroside.

In neuronopathic forms of GD (nGD), GCase activity is minimal or absent, insufficient to degrade even small amounts of endogenous substrate present in the central nervous system (originating from gangliosides synthesized in neurons). Undegraded glucocerebroside, deposited mainly in the endoplasmic reticulum, ultimately leads to neuronal damage and death.

GD is classified into three types depending on the presence of neurological symptoms. The non-neuronopathic form, known as type I (characterized by hepatosplenomegaly, thrombocytopenia, anemia, and bone involvement), is distinguished from the neuronopathic forms, which include type II (acute neuronopathic, also called infantile) and type III (subacute neuronopathic, also called juvenile). These are characterized by central nervous system (CNS) involvement (to varying degrees), as well as hepatosplenomegaly, hematologic manifestations.

Insufficient activity of the enzyme GCase is determined by pathogenic variants in the *GBA1* gene. To date, more than 600 *GBA1* pathogenic variants have been described; the most common are c.1226A>G, p.Asn409Ser (N370S) and c.1448T>C, p.Leu483Pro (L444P), which occur with different frequencies in selected populations [2]. Despite the recurrence of the most frequent *GBA1* variants, Gaucher disease exhibits a highly heterogeneous phenotype both within the neuronopathic forms (where a continuum between types II and III can be observed) and the non-neuronopathic form [3]. The presence of the N370S variant on one *GBA1* allele provides sufficient residual GCase activity to protect the CNS and clinically manifests as the non-neuronopathic type I disease. In patients with type 1 GD, despite the lack of neurological symptoms, the risk of developing Parkinson’s disease symptoms increases with age. Homozygosity for L444P leads to type III neuronopathic Gaucher disease (GD3) with varying severity of neurological symptoms [4].

Type III Gaucher disease (subacute neuronopathic,) is characterized by the onset of visceral and neurological symptoms during childhood, although neurological manifestations may develop later. Initially, CNS involvement presents as abnormal horizontal eye movements, known as saccadic (jerky) movements. As the disease progresses, a wide range of neurological symptoms develops, including tremors, myoclonus, myoclonic epilepsy, dysarthria, impaired muscle coordination, ataxia, progressive kyphosis (develops clearly during puberty), intellectual disability, and slowly progressive dementia. The course of neurological symptoms in GD3 is individual [3]. In these patients, the most common reported genotypes are L444P/L444P or L444P/D409H [5].

Enzyme replacement therapy (ERT) has been shown to normalize organ volumes, hematologic parameters, and biomarkers; however, it has no effect on the progression of neurological symptoms [6]. Ambroxol (ABX) has been studied in patients with nGD since 2016. Early reports [7] showed its safety, increased GCase activity, reduced Lyso-Gb1, and marked neurological improvement, including resolution of myoclonic seizures. Later studies confirmed variable outcomes depending on *GBA1* genotype: inconsistent in L444P homozygotes [8], but positive in other variants [9,10,11]. In GD-associated Parkinson’s disease (GD-PD), ABX was well tolerated and boosted GCase activity, however did not demonstrate clinical improvement in the treated patients [12]. Cell studies showed reduced sphingolipid accumulation and increased lysosomal translocation of GCase, with stronger effects in L444P carriers [13].

### 1.1. Characteristics of the Polish Patient Population

The clinical presentation and disease course in Polish patients with GD3 resemble the Norrbottnian phenotype observed in the Swedish population [14,15]. This phenotype is characterized by a marked splenomegaly and hepatomegaly in childhood, accompanied by less pronounced neurological symptoms that appear later and progress more slowly. Polish GD3 patients exhibit a phenotype with relatively mild neurological manifestations slowly progressing—intellectual development remains within the normal range. All patients with GD type 3 under our care (The Children’s Memorial Health Institute, CMHI, Warsaw, Poland) received ERT, which controls visceral symptoms as well as hematological parameters and biomarker levels, leading to normalization in most cases.

In the group of GD3 patients (total number: 13) under CMHI constant care (1980–2014), ABX was recommended as an off-label product at a proposed fixed dose of approximately 10 mg/kg/day. The treatment was safe and well tolerated. All Polish GD3 patients were monitored for neurological symptoms using the modified severity scoring tool for neuronopathic GD (mSST) [16,17].

In the Polish GD3 population, where all patients carry the homozygous L444P *GBA1* variant, clinical status varied. This fact provided the rationale for undertaking the present study, aimed at more detailed assessment of the efficacy of ambroxol in Polish GD3 patients.

### 1.2. Treatment

To date, no “targeted” therapy addressing neurological symptoms in GD has been available. New approaches are being developed, including other small molecule chaperones and gene therapy.

The currently applied approach aims to reduce glucocerebroside storage in macrophages. The alleviation of visceral and hematological symptoms in GD1 and GD3 relies on the uptake of recombinant enzyme into macrophages via the M6P receptor. The pathomechanism of CNS involvement in GD3 is entirely different—here, the disease process begins earlier, already in fetal life, where the absence of residual GCase activity results in the inability to degrade endogenous substrate (derived from gangliosides). However, in the case of CNS, GCase application is hindered by its inability to cross blood–brain barrier (BBB).

There are two main approaches to reducing the accumulation of glucosylceramide in the body and thereby mitigating the clinical manifestations of the disease:

- Enzyme replacement therapy (ERT): designed to enable macrophage-lineage cells to degrade glucosylceramide.
- Substrate reduction therapy (SRT): slows the accumulation of glucosylceramide by decreasing its synthesis.

### 1.3. Rationale for Clinical Study and Perspectives Related to ABX

As indicated by the studies discussed above, the use of ABX in the treatment of neurological complications associated with GCase deficiency appears promising. This applies both to patients with neuronopathic GD, where ERT is ineffective for neurological symptoms due to the BBB, and to the risk of PD associated with *GBA1* variants. However, questions remain regarding the long-term safety and efficacy of ABX, as well as the mechanisms underlying treatment response. This is due to several factors:

- No clinical trials have yet analyzed ABX efficacy in larger and more homogeneous cohorts of patients with neuronopathic GD; most reports describe single cases. Broader observational data on ABX safety and efficacy in GD patients with neurological symptoms and in at-risk groups (e.g., carriers) are, therefore, needed.
- Clinical assessment of neurological manifestations and severity is difficult and poorly comparable across studies. Patients were evaluated in different centers by different physicians, without a standardized assessment tool such as the modified severity scoring tool (mSST). Use of a standardized clinical assessment form for ABX-treated patients would improve comparability.

## 2. Materials and Methods

### 2.1. Patients

Initially, nineteen patients with type 1 and type 3 GD were eligible for the study, including 15 with type 3 GD and 4 with type 1 GD. However, 2 patients with type 3 and 4 patients with type 1 GD withdrew from the study due to personal reasons. Finally, the full study was initiated and completed by 13 patients with type 3 GD, L444P homozygotes, as described in the Introduction.

### 2.2. Ambroxol Dosing

The 10 mg/kg/day dose was selected and consisted of oral administration of ABX divided into three equal doses, for a duration of 360 days.

### 2.3. Primary Objectives of Clinical Trial

- Assessment of the clinical status of patients with type 3 GD using the mSST neurological symptom scale on ABX administration during the one-year study period.
- Evaluation of biomarkers (chitotriosidase, Lyso-Gb1) and hematological and biochemical parameters.
- Assessment of drug safety.

### 2.4. Study Design

The study was open-label without randomization. Each patient was assigned a unique identification number (two digits for the study center and two digits for the patient, e.g., 01-01).

The study group consisted of patients with neurological symptoms in the course of GD3, in whom ABX was either: (1) used previously in the dosage below 10 mg/kg/day and discontinued for at least 30 days, or (2) never used before. In this group, ABX was reintroduced for a period of 360 days after a 30-day discontinuation.

Patients were assessed during six visits (Visits 1–5) at the CMHI center.

### 2.5. Clinical Assessments and Study Procedures

Clinical status was evaluated (based on the diagnostic mSST test), together with biomarker assessments and hematology. The multidisciplinary team included a neurologist, psychologist, and physiotherapist. The mSST was conducted by a specialist in metabolic diseases.

After the 30-day discontinuation period, patients underwent the following examinations: mSST test, biomarker concentrations (including chitotriosidase and Lyso-Gb1), and hematology parameters (complete blood count, AST, ALT, ferritin, platelets, INR, creatinine).

Study procedures included the following:

- Clinical examination performed by a multidisciplinary team (Visits 1–5).
- Collection of blood and urine samples for the analyses.
- Basic laboratory tests (Visits 1–5)—complete blood count, aspartate aminotransferase (AST), alanine aminotransferase (ALT), ferritin, INR, creatinine, pregnancy test from urine and blood (for women of childbearing age).
- Biomarkers (Visits 1–5)—chitotriosidase, Lyso-Gb1.

Clinical assessment of the patients included:

- Medical history (sex, current age, genotype, age at diagnosis, age at initiation of enzyme replacement therapy (average doses), duration of treatment, current ERT dose, spleen preserved yes/no (age at splenectomy), current hematological results—hemoglobin concentration, platelet count, biomarkers—chitotriosidase, Lyso-Gb1, and bone densitometry).
- Physical examination, basic vital signs, anthropometry.
- Medical history of epilepsy course and treatment.
- mSST scoring based on current neurological examinations and medical records (retrospective).

### 2.6. mSST

Patients were clinically assessed for the presence of neurological symptoms using the modified severity scoring tool (mSST), commonly applied in patients with neuronopathic Gaucher disease [16,17].

The mSST is a test covering 11 groups of neurological symptoms observed in nGD. Scores range from 0 to 33 points, depending on the severity of clinical manifestations [16,17].

### 2.7. Biochemical Markers

The following indicators of metabolic changes were analyzed:

- Chitotriosidase activity;
- Lyso-Gb1 concentration.

Chitotriosidase is an enzyme produced by activated macrophages (loaded with unmetabolized substrate), to which certain bactericidal properties are attributed. In patients newly diagnosed with GD, chitotriosidase activity in serum is typically 100–4000 times higher compared to healthy individuals. Chitotriosidase is currently the most frequently measured biomarker in GD, and its activity correlates with disease severity, reaching higher values in more advanced clinical forms. Monitoring chitotriosidase levels in GD is also used to track treatment response and to assess GD activity after discontinuation of therapy [18].

Glucosylsphingosine (GlcSph, Lyso-Gb1) is a product of deacylation resulting from disturbed glycolipid homeostasis in GD [19]. It is a relatively new biomarker measured in dried blood spots (DBS) and used in the diagnosis and monitoring of GD patients [20]. Lyso-Gb1 levels in DBS were measured using high-pressure liquid chromatography–tandem mass spectrometry at Centogene, Rostock, Germany [21,22].

## 3. Results

### 3.1. mSST Scoring

**Figure 1:**
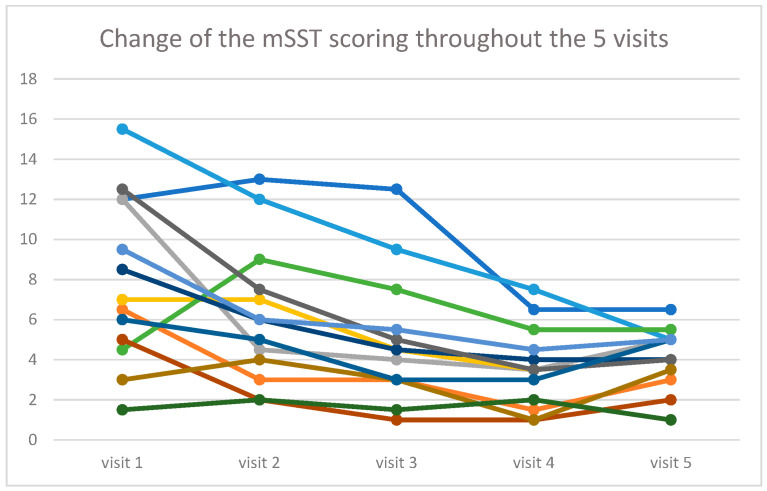
Time evolution of the mSST scoring during the five consecutive visits for all 13 patients. Each color represents a different patient.

**Figure 2:**
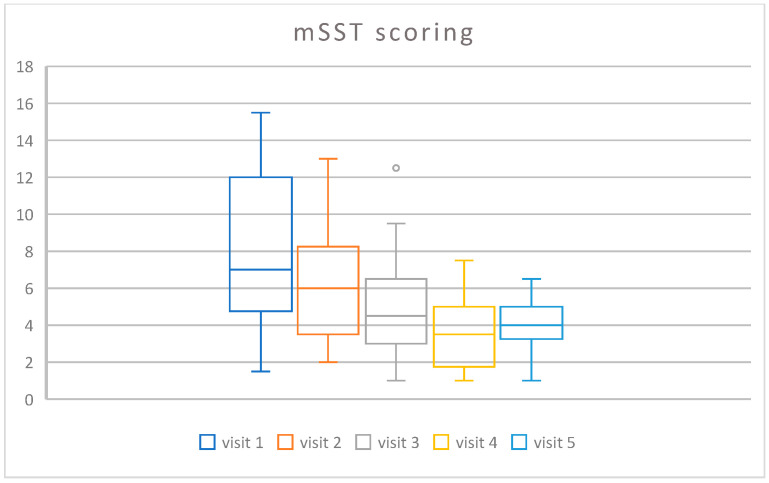
Box plots presenting the change in time of the treatment of the mSST scoring.

The Friedman ANOVA showed that the scoring diminished during the time of the treatment, from a median value of 7.0 at the beginning of the study to 4.5 at the end (chi = 19.34, *p* < 0.001). The spaghetti plot of all results is shown in Figure 1, and a box and whisker plot in Figure 2.

### 3.2. Hematology Assessment

There were no statistically significant changes in the hematology parameters, such as hemoglobin, hematocrit, leucocytes, lymphocytes, monocytes, neutrophils, eosinophils, basophils, AST, ALT, ferritin, platelets, and creatinine. The parameters in which statistically significant differences were found were erythrocytes (chi = 11.26, *p* = 0.0238) and INR (chi = 18.48, *p* < 0.001). The spaghetti plot of all patients’ results is shown in the Supplementary Material on Figure S1 (for erythrocytes) and Figure S3 (for INR), and box and whisker plots on Figure S4 (for erythrocytes) and Figure S6 (for INR).

### 3.3. Biochemical Markers

**Figure 3:**
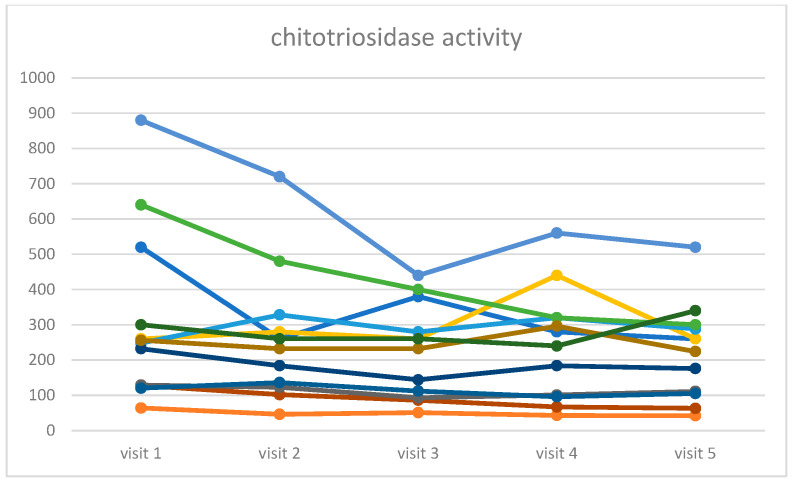
Time evolution of the chitotriosidase activity recorded during the five consecutive visits for the cohort of 12 patients (one outlier omitted for clarity). Each color represents the different patient.

**Figure 4:**
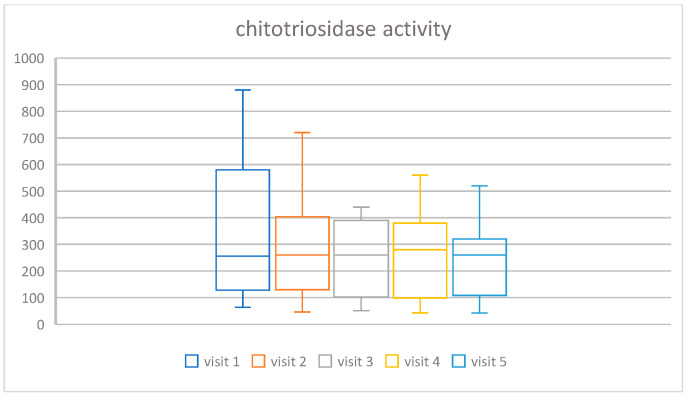
Box plots presenting the change in time of the treatment of chitotriosidase activity for the cohort of 12 patients (one outlier omitted for clarity).

**Figure 5:**
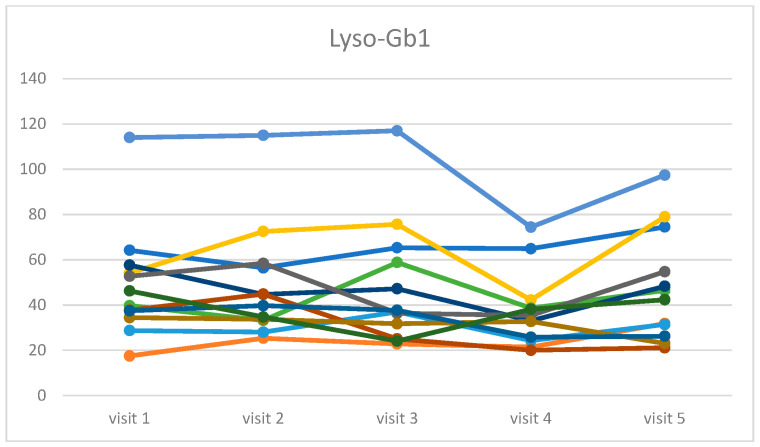
Time evolution of the Lyso-Gb1 concentration recorded during the five consecutive visits for the cohort of 12 patients (one outlier omitted for clarity). Each color represents the different patient.

**Figure 6:**
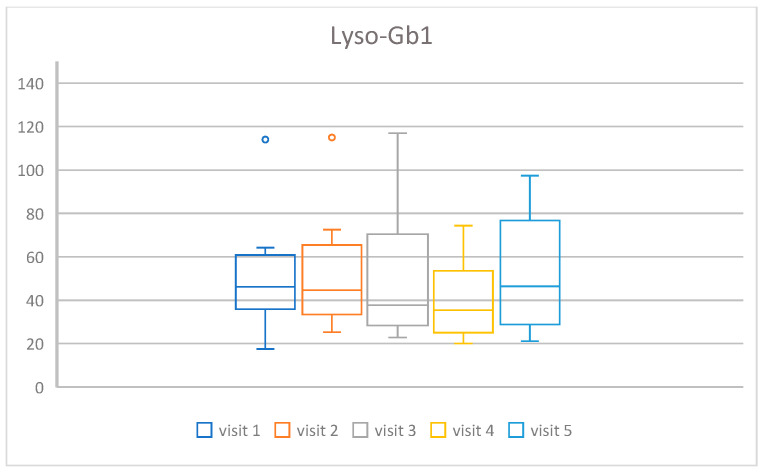
Box plots presenting the change in time of the treatment of Lyso-Gb1 concentration for the cohort of 12 patients (one outlier omitted for clarity).

The following indicators of metabolic changes were analyzed: chitotriosidase activity and Lyso-Gb1 concentration. In both markers, statistically significant changes were found: chi =16.62, *p*-value = 0.0023 for chitotriosidase activity, and chi = 11.45, *p*-value = 0.022 for Lyso-Gb1 concentration. In Figure 3 and Figure 4, we present the spaghetti plot and box and whisker plot for chitotriosidase activity, and an analogous visualization in Figure 5 and Figure 6 for Lyso-Gb1. Please note that for all four figures, one patient was removed from the cohort for the clarity of the plot and in order to maintain a readable scale (his results were very high throughout a 30-year ERT, despite a very high treatment dose—60 u/kg/eow (personal observations)). The set of figures containing the results for all 13 patients is in the Supplementary Material.

### 3.4. Safety Analysis

During the study, no adverse events related to the study drug were observed, while one patient (5%) met the SAE criterion due to an event. Three other events occurred in three other patients (15%)—in one patient it was a skin allergic reaction in the form of urticaria, in two patients it was nausea.

Other reactions included:

- Migraines—occurred in six patients (30%);
- An episode of anxiety requiring hospitalization, resulting in discontinuation of the drug during hospitalization—one patient (5%);

## 4. Discussion

Over a one-year observation period, 13 patients with GD3 receiving ABX at 10 mg/kg/day as an adjunct to ERT demonstrated a good tolerability, with no adverse events reported. Neurological assessment using the mSST revealed a clinically meaningful improvement, including a reduction or complete cessation of epileptic seizures in previously symptomatic individuals. Tremor intensity decreased or resolved, and tendon reflexes normalized. Overall, patients exhibited cognitive improvement, reflected in enhanced performance at work and in daily functioning.

Although multiple case reports have suggested beneficial effects of ABX in reducing the severity of neurological symptoms, prior observations were limited to isolated cases and lacked standardized assessment tools such as the mSST [23]. The original Narita paper was limited by the multi-center approach and the limited number of patients but did report long-term follow up in excess of 12 months. The present findings, therefore, provide the supportive evidence from a larger, systematically evaluated patient group. The most important finding was the improvement of the functional status of the patients reflected by the systematic change in time in the mSST score.

Chitotriosidase activity and Lyso-Gb1 levels decreased somewhat compared to the level before using ABX, and these were statistically significant changes. The explanation for the decrease in the two biomarkers chitotriosidase and Lyso Gl1 may be related to the fact that ABX, which increases GBA activity (as a chaperone or otherwise), also acts on the native enzyme at the “periphery” in patients homozygous for L444P. This was clearly noticeable in our study, but the ABX dose was relatively low compared to other reported studies (10 mg/kg/day or bw).

Other biochemical (AST, ALT) and hematological parameters (except erythrocytes and INR) showed no significant changes.

Since its discovery as a chaperone in 2009, the efficacy of ABX in the treatment of patients with GD was reported for ABX in combination with the currently available ERT/SRT treatment and also when ERT or SRT was not available or affordable for some patients. Several overviews of clinical studies on the efficacy and safety of ABX in GD have been published, thus we want to underline the recent ones.

Istaiti et al. [24] described the efficacy of 12 months of 600 mg ABX as an adjuvant therapy to ERT or SRT in a homogeneous cohort of patients with GD1 (22 heterozygous for N370S *GBA1* variant) who showed poor or no response to these specific modalities. Of the six patients who had entered the study from the thrombocytopenia group, three achieved the primary outcome of greater than 20% improvement in platelet counts. Of the 11 patients who had entered the study from the DEXA group, five achieved the primary outcome of greater than 0.2 improvement in lumbar spine T-score. Of the eight patients who had entered the study from the Lyso-Gb1 group (six from the ERT or SRT group and two from the naïve group), two patients achieved a greater than 20% reduction in Lyso-Gb1 levels.

Zhan et al. [25] reported the results of 28 patients (most with GD1) without GD-specific treatments treated with an escalating dose of ABX (mean [SD] dose, 12.7 [3.9] mg/kg/d) for a mean (SD) period of 2.6 (1.7) years. An increase in hemoglobin level was observed 1 year after the initiation of ABX. Reduction in liver and spleen volume was observed; the mean spleen volume decreased by 29.5% and the mean liver volume decreased by 21.1%. Reductions in Lyso-Gb1 level and chitotriosidase activity occurred after the initiation of ABX, and the reductions were maintained. The results are promising, however most of the 26 individuals in the study who were considered good responders to ABX carried mild variants, including N370S.

A major limitation of ABX efficacy in patients with GD is the selection of the proper ABX dosage. In the literature, ABX doses range from 5–25 mg/kg/day. Pilot studies reported neurological improvement within 3–6 months at doses of 9, 12, and 25 mg/kg/day [7], or after 6 months at doses as low as 1.5–3.5 mg/kg/day [26]. The selected dose (10 mg/kg/day) of ABX in our study is safe and appears sufficient to assess omic-level effects, while optimizing capsule intake. In a study authored by Istaiti et al. [24], maximum doses reach 600 mg/day in adults, which at 60 kg body weight corresponds to 10 mg/kg/day. The results obtained by authors strongly showed that 600 mg/day ABX dose might be efficient for most affected patients with GD.

The studies authored by Istaiti et al. [24] and Zhan et al. [25] proved the efficacy of ABX in quite homogeneous populations of patients with GD1 in Israel and China. In our case, we provide for the first time the results of 12-month ABX treatment of patients with GD3, showing its efficacy in the context of neurological symptoms.

## 5. Conclusions

The results presented in 13 Polish patients with GD3 who were treated with a dose of 10 mg/kg of ambroxol for one year showed neurological improvement, as assessed by the mSST. Some reductions in the levels of two specific biomarkers, chitotriosidase and Lyso-Gb1, were also demonstrated.

A dose of 10 mg/kg of ABX was effective not only in reducing the expression of neurological symptoms, but also the levels of chitotriosidase and Lyso-Gb1, and was free of side effects and safe to use.

The analysis of adverse events did not reveal any events related to the therapy used in patients.

## Data Availability

The data supporting the findings of this study are not openly available due to sensitivity and privacy concerns. However, they are available from the corresponding author on reasonable request, provided that the request complies with relevant ethical guidelines and data protection regulations. The data are the intellectual property of the study Sponsor and Medical Research Agency, and will be made available with their consent.

## Supplementary Materials

The following supporting information can be downloaded at: https://www.mdpi.com/article/10.3390/life16030485/s1, Figure S1. Time evolution of the erythrocytes percentage of the maximum reference value during the consecutive five visits, for all 13 patients; Figure S2. Box-plot presenting the change in time of the treatment of erythrocytes. The Y axis shows the % of erythrocytes in respect to the maximum reference value; Figure S3. Time evolution of the INR value recorded during the consecutive five visits, for all 13 patients; Figure S4. Box-plot presenting the change of INR (International Normalized Ratio) during the treatment; Figure S5. Time evolution of the chitotriosidase activity recorded during the consecutive five visits, for all 13 patients; Figure S6. Box-plot presenting the change of chitotriosidase activity during the treatment, for all 13 patients; Figure S7. Time evolution of the Lyso-Gb1 concentration recorded during the consecutive five visits, for all 13 patients; Figure S8. Box-plot presenting the change of Lyso-Gb1 concentration during the treatment, for all 13 patients; Table S1. Results of the mSST score for all 13 evaluated patients, broken down into assessed components. The five numbers in each block represent five scores obtained during consecutive visits for a mSST element (column) by each patient (row).

## Abbreviations

The following abbreviations are used in this manuscript:

ABX	Ambroxol
AE	Adverse events
ER	Endoplasmic reticulum
ERT	Enzyme replacement therapy
*GBA1*	Gene encoding glucocerebrosidase
GBA-PD	Parkinson’s disease associated with GBA variants
GD	Gaucher disease
GD3	Type III Gaucher disease
GD1-PD	Type I Gaucher disease with increased risk of Parkinson’s disease
INR	International normalized ratio
Lyso-Gb1/Lyso-GL1	Glucosylsphingosine
nGD	Neuronopathic Gaucher disease
PCT	Pharmacological chaperone therapy
PD	Parkinson’s disease
