# Supplementary material for: Safety and Efficacy of Ambroxol Therapy in Polish Patients with Gaucher Disease
Source: Life (Basel). 2026 Mar 16;16(3):485. doi: 10.3390/life16030485 (PMC13028086; doi:10.3390/life16030485)
Supplement: Supplementary file 1 [file life-16-00485-s001.zip › life-4103123-supplementary.pdf]

Figure S1 Time evolution of the erythrocytes percentage of the maximum reference value during the consecutive five visits, for all 13 patients.

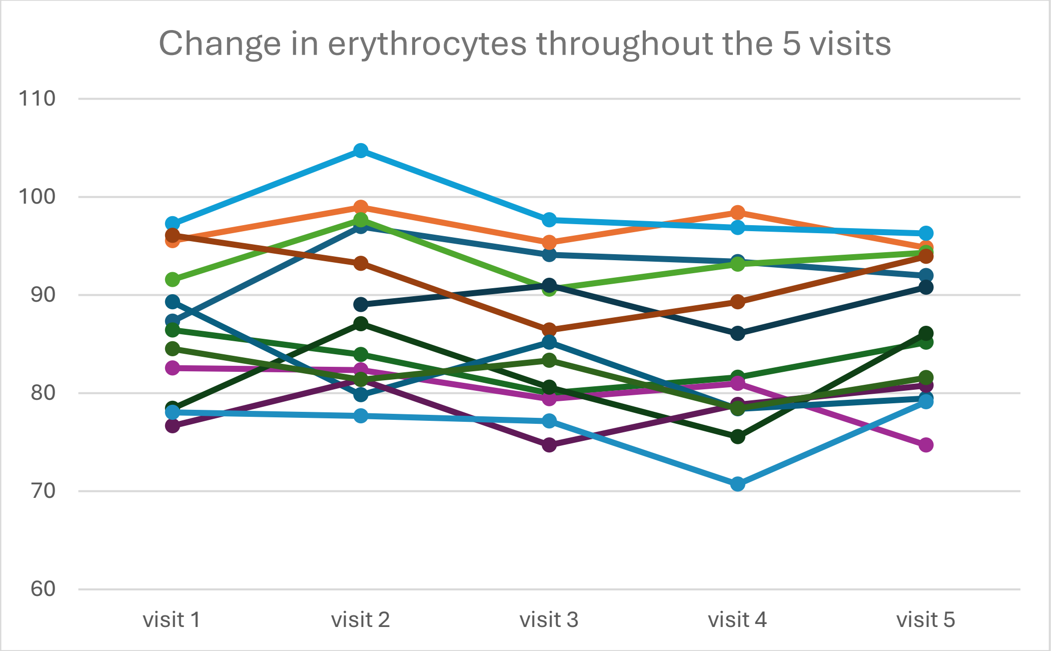

Figure S2. Box-plot presenting the change in time of the treatment of erythrocytes. The Y axis shows the % of erythrocytes in respect to the maximum reference value.

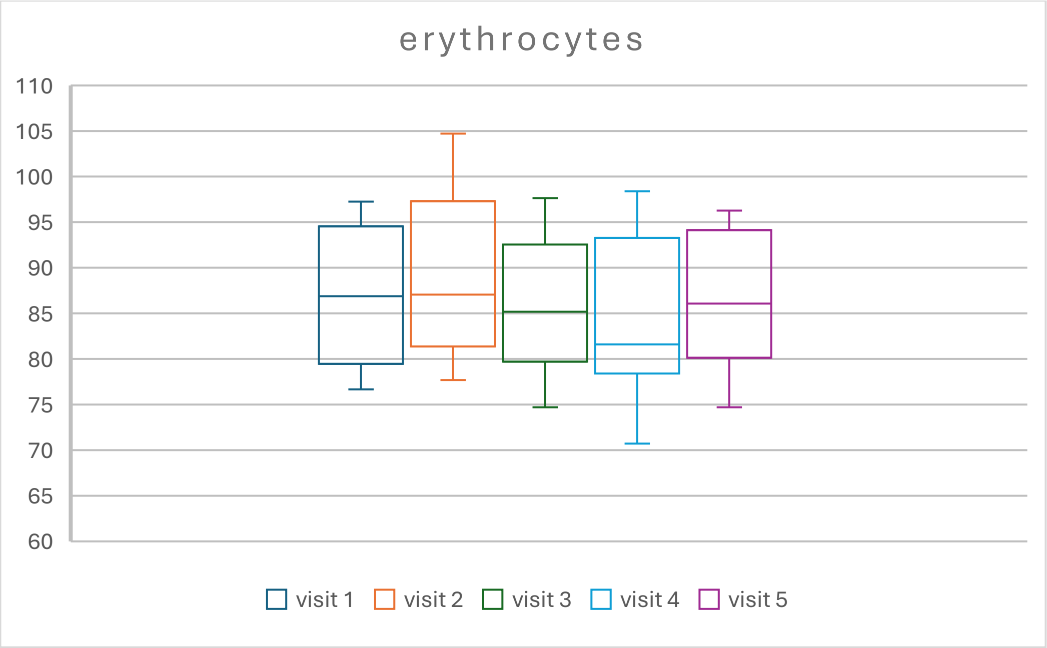

Figure S3. Time evolution of the INR value recorded during the consecutive five visits, for all 13 patients.

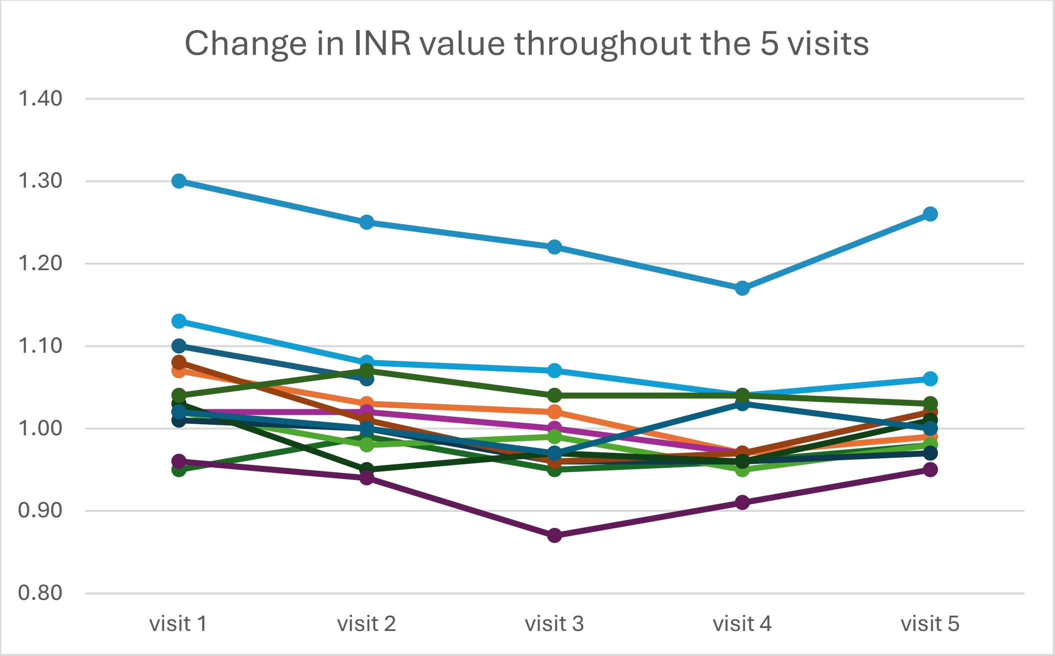

Figure S4. Box-plot presenting the change of INR (International Normalized Ratio) during the treatment.

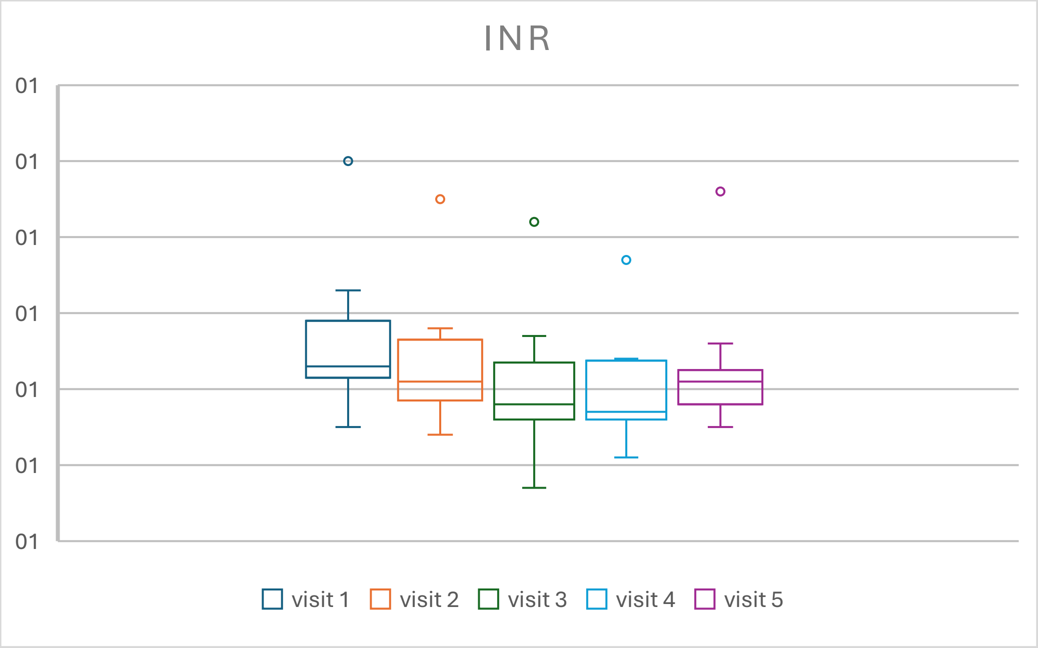

Figure S5. Time evolution of the chitotriosidase activity recorded during the consecutive five visits, for all 13 patients.

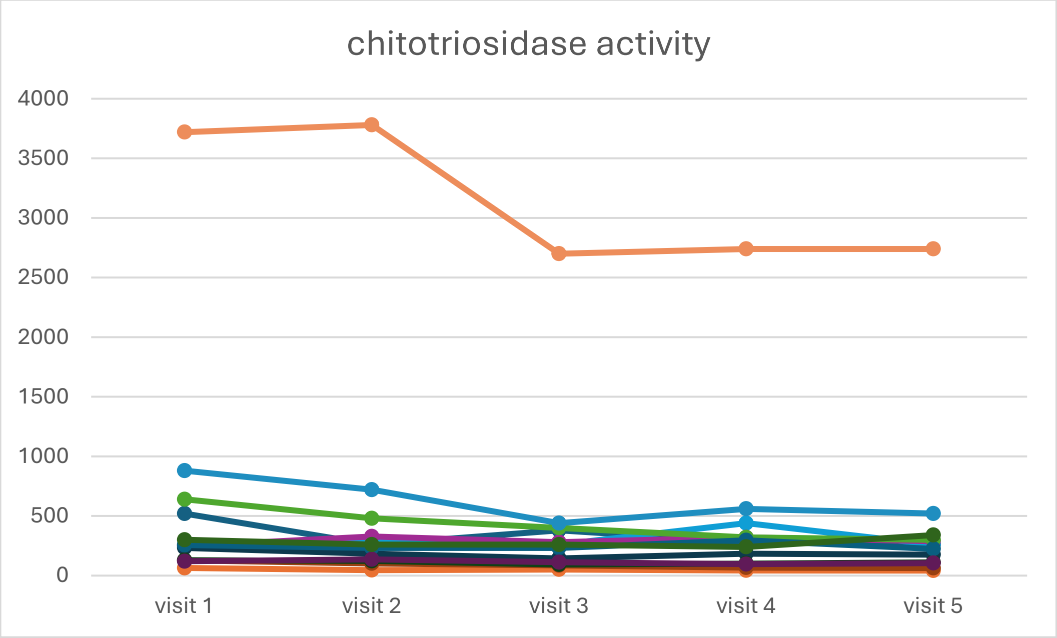

Figure S6. Box-plot presenting the change of chitotriosidase activity during the treatment, for all 13 patients.

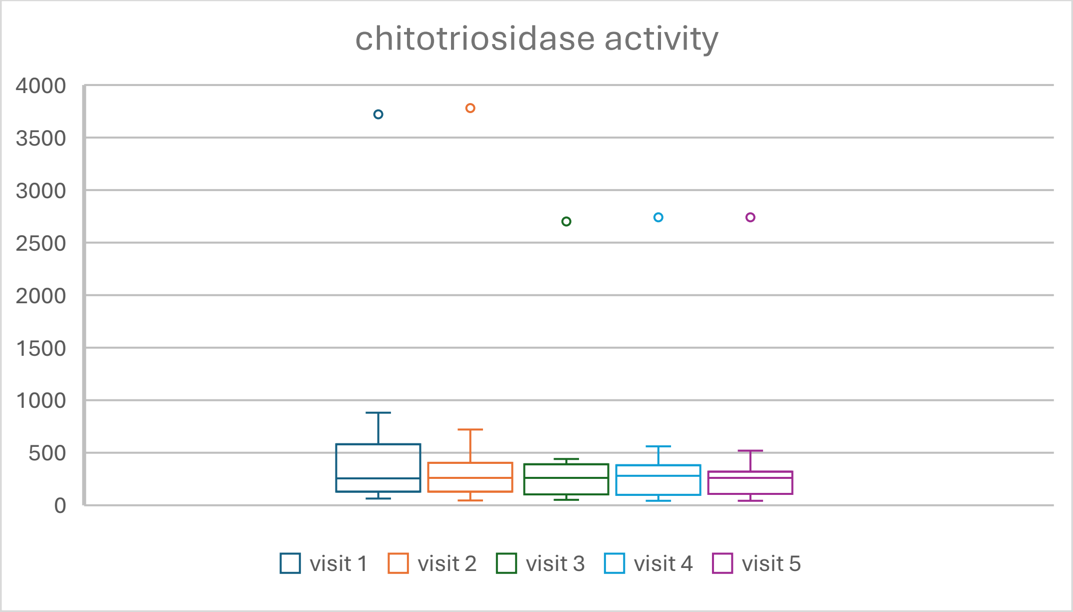

Figure S7. Time evolution of the Lyso-Gb1 concentration recorded during the consecutive five visits, for all 13 patients.

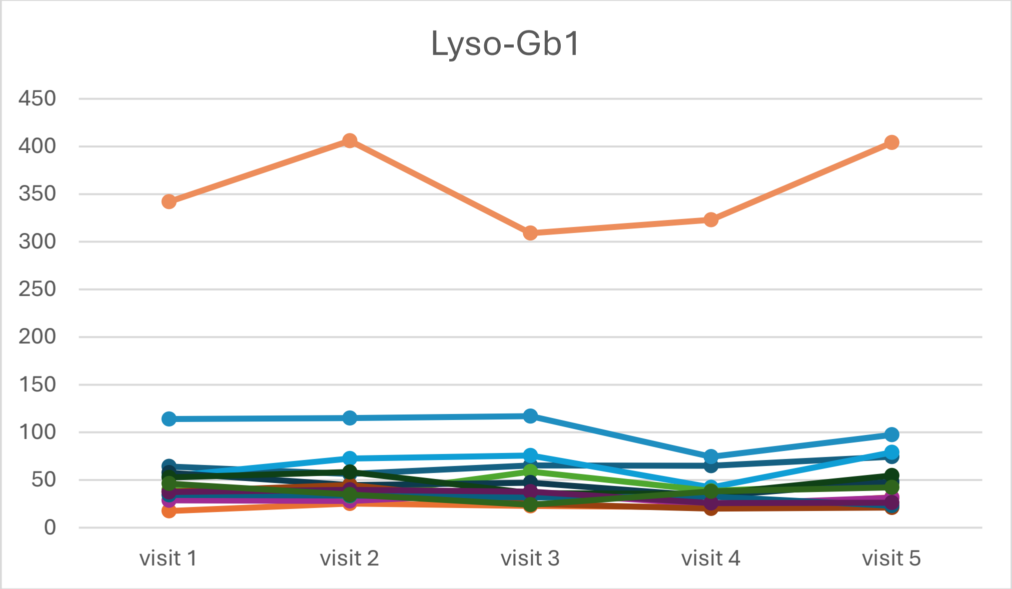

Figure S8. Box-plot presenting the change of Lyso-Gb1 concentration during the treatment, for all 13 patients.

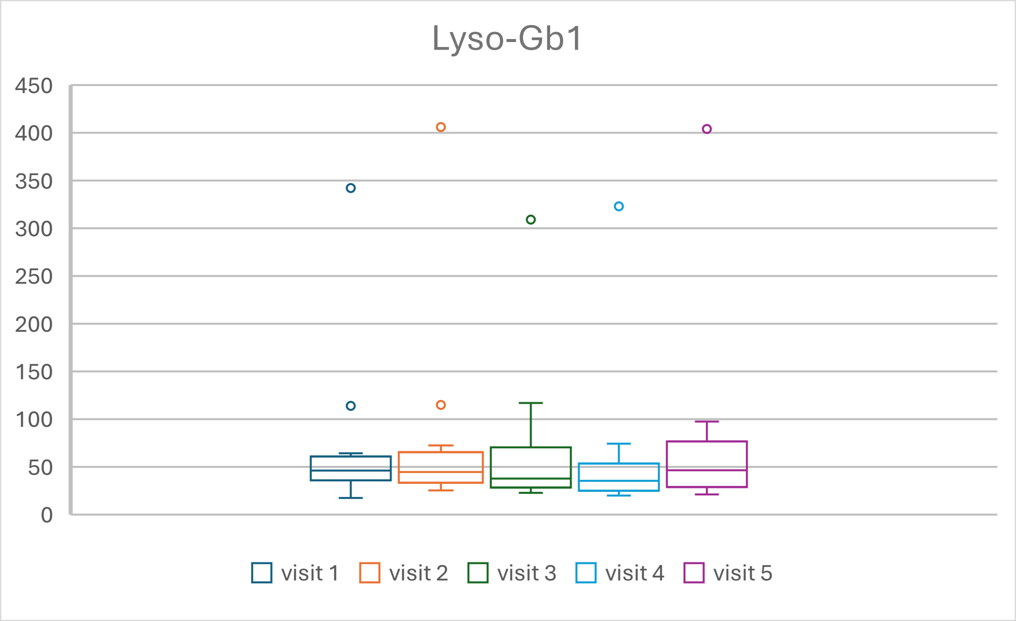

	mSST												
	oculomotor apraxia	seizures	mental retardation	ataxia	Cerebellar features	Pyramidal features	Extrapyramidal features	Bulbar symptoms/difficulty swallowing	speech disorders	fine motor skills	gross motor skills	ophthalmological features	kyphosis
1	1.5,1,1,0,1	0,0,0,0,0	1,0,0,0,0	1.5,1,0,0.5,0	0,0,0,0,0	1,0,0,1,0	0,0,0.5,1,0.5	0,0,0,0,0	1,1,1,0.5,1	0,0,0,0,0	0,0,0,0,0	1.5,0,0,0,0	3,3,3,3,3
2	0,0,0.5,0,0.5	0,0,0,0,0	1,1,1,0.5,0.5	0,0,0,0,0	1.5,0,0,0,0	1,0,0.5,0,0	1,1,0,0,0	0,0,0,0,0	1,1,0,0.5,0	0,0,0,0,0	0,0,0,0,0	0,0,0.5,0,0	3,3,2.5,3,3
3	0,0,0,1,0	0,0,0,0,0	1,1,0,0,0.5	1,1,0,0,0	3,0.5,0,1,1	1,0.5,0,0,0	1,1,0,1,0	0,0,0,0,0	1,1,1,0.5,0.5	0,0,0,0,0	2,2,0,0,0	0,0,0,0,0	3,3,3,3,3
4	1.5,0,1,0,0	0,0,0,0,0	0,0,0,0,0	0,0,0,0,0	0,0,0,0,0	0,0,0,0,0	0,1,0,0,0	0,0,0,0,0	0,0,0,0,0	0,0,0,0,0	0,0,0,0,0	0,0,0,1,0	2,1,1.5,1,1
5	1.5,0,0,0,0.5	0,0,0,0,0	0,0,0,0,0	0,0,0,0,0	1.5,0,0,0,0	0,0,0,0,0	0,0,0,0,0.5	0,0,0,0,0	0,0,0,0,0	0,0,0,0,0	0,0,0,0,0	0,0,0,0,1.5	3,3,3,3,3
6	0,0,0,0,0	0,0,0,0,0	2,1,1,1,1	0,0,0,0,0	0,0,0,0,0	1,0,0,0,0	0,0,0,0,0	0,0,0,0,0	1,0,0,0,0	0,0,0,0,0	0,0,0,0,0	0,0,0,0,0	1,1,1,0,1
7	0,0,0,0,0	2,0,0,0,0.5	1,0,0,0,0.5	0,0,0,0,0	1.5,0,0,0,0	1,1,0,0,0	0,0,0,0,0	0,0,0,0,0	0,0,1,0.5,0.5	0,0,0,0,0	0,0,0,0,0	0,0,0,0,0	2,2,2,1,1.5
8	1.5,1.5,0.5,0,0.5	3,2,0.5,0.5,1	1,1,0.5,1,2	0,0,0,0,0.5	1.5,0,0,0,0	1,1,0,0,0	0,0,0,0,0.5	0,0,0,0,0	1,1,0,0,0.5	0,0,0,0,0	0,0,0,0,0	0,0,0.5,0,0	2,2,3,2,2
9	1,0,0,0,0	0,0,0,0,0	0,0,0,0,0	0,0,0,0,0	1,1,0,0,0	0,0,0,0,0.5	0,0,0,0,0	0,0,0,0,0	0,0,0,0,0	0,0,0,0,0	0,0,0,0,0	0,0,0,0,0	3,3,3,1,3
10	1.5,1.5,1.5,1.5,1	0,0,0,0,1	1,1,0.5,0.5,0.5	0,0,0,0,0	1.5,0,0,0,0	1,1,0,0,0	0,0,0,0,0	0,0,0,0,0	1,1,0.5,0.5,0	0,0,0,0,0	0,0,0,0,0	0,0,0,0,0	3,3,3,3,3
11	1.5,1.5,1,0,1	3,2,1,2,1	1,1,0.5,0.5,0	0,0,0,0,0	1.5,1,0,0,0	1,1,0,1,0	1,1,0,0,0.5	1,1,0,0,0	1,1,1,1,0.5	0,0,0,0,0	0,0,0,0,0	0,0,0,0,1.5	3,3,3,3,3
12	1.5,1.5,1,1,1.5	2,0,1.5,1,1	0,0,0,0,0	0,0,0,0,0	1.5,1,0,0,0	1,1,0,0,0	1,1,0,0,1	1,1,0,0,0	1,1,1,1,1	0,0,0,0,0	0,0,0,0,0	0,0,1,0,0	3,3,3,3,3
13	1.5,0.5,0,1,1	0,0,0,0,0	0,0,0,0,0	0,0,0,0,0	1.5,0,0,0,0	1,1,0.5,0,0	0,0,0,0,0	0,0,0,0,0	0,0,0,0,0	0,0,0,0,0	0,0,0,0,0	0,0,0,0,0	3,3,3,3,3

Table S1. Results of the mSST score for all 13 evaluated patients, broken down into assessed components. The five numbers in each block represent five scores obtained during consecutive visits for a mSST element (column) by each patient (row).
